# Excision of the Mamma under Hypnotic Anæsthesia

**Published:** 1890-09-13

**Authors:** 


### SURGERY.

### EXCISION OF THE MAMMA UNDER HYPNOTIC ANÆSTHESIA.

While the practice of hypnotism by non-medical persons, its exhibition as a public entertainment, and its employment, even by medical men, without a definite purpose for the relief of the individual subject should be as absolutely put down as would similar performances with ether or chloroform, it would be to repeat an error to which the subsequent discredit and degradation of the procedure is in great measure due, were hypnotism to be declared illegal under all circumstances, or were the medical profession to condemn it untried, as they did half-a-century ago when Drs. Esdaile, Elliottson, Inglis, &c., first availed themselves of it, and endeavoured to investigate its value as a therapeutic agent. Dr. Esdaile performed over three hundred capital operations, including lithotomy, amputation of the thigh, and excision of the elephantiasis tumours at Calcutta, under conditions of total anæsthesia, while chloroform, &c., were, as yet, unknown.

Dr. Schmeltz, of Strasburg, having been consulted by a young lady, aged 20, about a tumour of one breast, which appeared, and ultimately proved, to be malignant, the axillary glands being still uninvolved, and her general health good, determined on removing the entire mamma. He observed that she could be hypnotised with remarkable ease, a fixed gaze, with a few passes of the hands, sufficing to induce the hypnotic state, and so soon as her eyes closed the supervention of anæsthesia to acupuncture, &c., and she expressed her willingness that this means should be employed in preference to chloroform. On the day fixed for the operation he found, however, that the mental emotion prevented the attainment of the desired degree of anæsthesia; and having given her to understand that it would be postponed for a week, he anæsthetised her successfully the next day, when she was not disturbed by apprehension. He was assisted by Drs. Janza and Barrier, who wished to have chloroform and ether at hand in case of failure, but this Dr. Schmeltz declined as unnecessary. During the operation she felt nothing, but was so far awake to the procedure that she held up her arm, turned from time to time into the most convenient positions, and talked and laughed about what was going on. He operated very leisurely, and when he had finished, told her to have a good night and to feel no pain, which she did, to the satisfaction of all concerned. Dr. Schmeltz remarks that hypnotism, when the patient is susceptible of it, presents this great advantage over chloroform, that the anæsthesia, though fully as complete, can be maintained so long as the operator desires. During the whole time the pulse did not vary in the least, proving the absence of pain, shock, or depression, and he calls attention to the importance of not allowing the patient to know the precise time when the operation is to be performed. It is true that this operation was not a difficult or grave one, but cutaneous sensibility is most acute; and since the fullest anæsthesia is attainable, it would appear that hypnotism might be employed with success and inestimable advantage in abdominal surgery, operations which are necessarily tedious, in which the danger of fatal depression and collapse is greatest, and of which the subjects are mostly women, and as such, usually most susceptible of hypnotic treatment.

